# The Chromosome-Level Genome Assembly of European Grayling Reveals Aspects of a Unique Genome Evolution Process Within Salmonids

**DOI:** 10.1534/g3.118.200919

**Published:** 2019-03-04

**Authors:** Tiina Sävilammi, Craig R. Primmer, Srinidhi Varadharajan, René Guyomard, Yann Guiguen, Simen R. Sandve, L. Asbjørn Vøllestad, Spiros Papakostas, Sigbjørn Lien

**Affiliations:** * Department of Biology, University of Turku, 20014 Turku, Finland; † Organismal & Evolutionary Biology Research Program, Faculty of Biological & Environmental Sciences; ‡ Institute of Biotechnology, University of Helsinki, 00014 Helsinki, Finland; § Department of Biosciences, University of Oslo, 0316 Oslo, Norway; ** INRA, UMR1313 GABI Génétique Animale et Biologie Intégrative, Domaine de Vilvert, 78352, Jouy-en-Josas Cedex, France; †† INRA, UR1037 Fish Physiology and Genomics, F-35000, Rennes, France; ‡‡ Centre for Integrative Genetics (CIGENE), Department of Animal and Aquacultural Sciences, Faculty of Biosciences, Norwegian University of Life Sciences, 1430 Ås, Norway

**Keywords:** chromosome evolution, chromosomal structure, genomic rearrangements, karyotype evolution, retrotransposons

## Abstract

Salmonids represent an intriguing taxonomical group for investigating genome evolution in vertebrates due to their relatively recent last common whole genome duplication event, which occurred between 80 and 100 million years ago. Here, we report on the chromosome-level genome assembly of European grayling (*Thymallus thymallus*), which represents one of the earliest diverged salmonid subfamilies. To achieve this, we first generated relatively long genomic scaffolds by using a previously published draft genome assembly along with long-read sequencing data and a linkage map. We then merged those scaffolds by applying synteny evidence from the Atlantic salmon (*Salmo salar*) genome. Comparisons of the European grayling genome assembly to the genomes of Atlantic salmon and Northern pike (*Esox lucius*), the latter used as a nonduplicated outgroup, detailed aspects of the characteristic chromosome evolution process that has taken place in European grayling. While Atlantic salmon and other salmonid genomes are portrayed by the typical occurrence of numerous chromosomal fusions, European grayling chromosomes were confirmed to be fusion-free and were characterized by a relatively large proportion of paracentric and pericentric inversions. We further reported on transposable elements specific to either the European grayling or Atlantic salmon genome, on the male-specific *sdY* gene in the European grayling chromosome 11A, and on regions under residual tetrasomy in the homeologous European grayling chromosome pairs 9A-9B and 25A-25B. The same chromosome pairs have been observed under residual tetrasomy in Atlantic salmon and in other salmonids, suggesting that this feature has been conserved since the subfamily split.

Whole genome duplication is known to be an important driver of evolutionary novelty and speciation (*e.g.*, Blomme *et al.* 2006; Van de Peer *et al.* 2009). Whole genome duplication is also regarded as a trigger of certain dramatic consequences in genome evolution (Lien *et al.* 2016). A tetraploid genome, for instance, is expected to be very unstable due to a variety of reasons including multivalent pairing during meiosis, unequal separation of sister chromosomes during mitosis, and gene dosage imbalances (Comai 2005; Edger and Pires 2009; Hufton and Panopoulou 2009). Chromosomal rearrangements, which are often associated with increased transposable element activity, are expected to be frequent during this period of genomic instability to restore a disomic inheritance of chromosomes, which is also called a rediploidization process (Ohno 1970; Semon and Wolfe 2007; Hufton and Panopoulou 2009; Lien *et al.* 2016). Also possibly driven by transposable element activity, chromosomal fusions, fissions, inversions and indels can suppress, for example, multivalent pairing, and they are also expected to lead to sequence divergence and genome evolution causing genomic incompatibilities among populations thus raising species barriers (Rieseberg 2001; Hoffmann and Rieseberg 2008; Makhrov 2017). To this end, sequencing and comparing the genomes of taxa with a recent common genome duplication event that have evolved radically different karyotypes holds the promise to illuminate questions regarding the evolutionary consequences of various types of chromosomal rearrangements (*e.g.*, Charlesworth 2016; Wellenreuther and Bernatchez 2018).

The Salmonidae family, also termed salmonid fish, represents an intriguing model system to study genome evolution following whole genome duplication. Salmonid fish have in common a whole genome duplication event that has occurred relatively recently, approximately 80-100 million years ago (Allendorf and Thorgaard 1984; Berthelot *et al.* 2014; Macqueen and Johnston 2014). It is considered that the hypothetical ancestor of salmonids had a typical diploid teleost genome with approximately 50 possibly acrocentric chromosomes, and thus the duplication event resulted in approximately 100 possibly acrocentric chromosomes with tetrasomic inheritance (Phillips and Ráb 2001). Recent evidence suggests that most of the diploid inheritance has been restored prior to lineage diversification, albeit some regions may still be under residual tetrasomy and thus recombining (Berthelot *et al.* 2014; Lien *et al.* 2016; Robertson *et al.* 2017).

Present-day salmonids have evolved drastically different karyotypes, which suggests the occurrence of very different genome evolution processes. Lineage diversification has resulted in three salmonid subfamilies: Thymallinae, which includes the European grayling (*Thymallus thymallus*); Coregoninae, which includes round whitefish (*Prosopium* spp.), whitefish and cisco (*Coregonus* spp. and *Stenodus* spp.); and Salmoninae, which is the subfamily of the well-studied Atlantic salmon (*Salmo salar*) as well as Pacific salmon and trout (*Oncorhynchus* spp.) (Phillips and Ráb 2001; Macqueen and Johnston 2014). These subfamilies are known to radically differ in the number of chromosomes and chromosomal arms (Phillips and Ráb 2001). The European grayling represents an extreme case as it has an exceptionally high number of chromosomes compared to other salmonids, between 2n = 98 and 2n = 102 depending on the subspecies. The number of European grayling chromosomes has thus remained approximately the same as the number of chromosomes from the ancestral salmonid genome straight after the salmonid-specific whole genome duplication (Phillips and Ráb 2001). European graylings also have an exceptionally high number of chromosomal arms, up to 170, which is considered to represent a marked increase over the assumed 100 arms of the hypothetical ancestral duplicated genome of salmonids (Phillips and Ráb 2001; Ocalewicz *et al.* 2013). This is assumed to be a consequence of pericentric inversions, that is, inversions containing the centromere of the ancestral acrocentric chromosomes (Phillips and Ráb 2001; Ocalewicz *et al.* 2013). The rest of the salmonid species have at least a third fewer chromosomes, with Atlantic salmon at the lower end of the distribution with a karyotype of n = 27 and n = 29 chromosomes in the North American and European clade, respectively, and the number of chromosomal arms as low as 72 (Phillips and Ráb 2001). Many Atlantic salmon chromosomes are also large and metacentric (from ssa01 to ssa07) or large and acrocentric (from ssa09 to ssa20) and are thought to have resulted from Robertsonian fusions of ancestral chromosomes, that is, a fusion of two acrocentric chromosomes at their centromeres (Phillips and Ráb 2001; Lien *et al.* 2016). As such, the Atlantic salmon and European grayling genomes represent clearly distinct genome evolutionary processes that have occurred within salmonids, which demands further investigation.

In this study, we report the first chromosomal-level genome assembly for the European grayling and its in-depth comparison with the Atlantic salmon genome. The assembly builds on the recently published scaffold-level assembly of European grayling that was assembled purely from short-read sequences (Varadharajan *et al.* 2018). Scaffold-level genome assemblies can provide excellent source materials for chromosomal-level assembly by employing additional data sources such as long reads, linkage mapping, and synteny with closely related species. Annotating and studying this new European grayling genome assembly further revealed novel insights into the genome evolution differences between the European grayling and Atlantic salmon.

## Material and Methods

### Assembling the European grayling genome at the chromosome level

#### Assembly of genomic scaffolds using long-read sequence data:

Using the PacBio RS2 platform, we sequenced the same DNA sample used in the recently published European grayling genome assembly (Varadharajan *et al.* 2018) at approximately 19x depth. The sample belonged to a single male adult fish caught from the River Glomma at Evenstad, Norway (61.42 N 11.09 E) that was killed in October 2012. The sequencing effort resulted in a total of 40 gigabase pairs of sequence information. PacBio reads with length >5 kilobase pairs were then processed to consensus sequences using the Canu assembler (Koren *et al.* 2017). The resulting reads with length >10 kilobase pairs, amounting to approximately 5x depth, were used in a hybrid assembly. The PacBio reads and the previously published Illumina-based assembly (Varadharajan *et al.* 2018) were merged together using the PBJelly2 suite (English *et al.* 2012) using the noSplitSubreads, minMatch 8, minPctIdentity 70, bestn 1 and maxScore 11 parameters. Basic statistics, such as N_50_, L_50_ and the length range of the assembled sequences were calculated for each assembly using an in-house developed script (contig_statistics.pl; available in GitHub). After initial mapping of the assembled scaffolds to the Atlantic salmon genome assembly (described in more detail in the linkage mapping section) and manual curation, some of the assembled scaffolds were split in cases of potential sequencing or assembly errors.

#### Linkage mapping:

Male- and female-based linkage maps were built using markers from a single European grayling family originating from the Rhine River (Obenheim, France) that included both parents, 69 female offspring and 44 male offspring that were sequenced using a restriction site associated DNA (RAD) methodology according to previously described protocol (Amores *et al.* 2011). The RAD fragments were produced by using the *Sbf*I restriction enzyme and were sequenced using 100 base pair single-end sequencing using the Illumina HiSeq 2500 platform. Quality trimming of the sequence reads was performed with ConDeTri v. 2.3 (Smeds and Kunstner 2011). The RAD data consisted of a total of 4,167,787 and 7,056,371 reads for the male and female parents, respectively, and an average of 4,041,607 reads for each offspring. Scaffolds containing at least one marker covered 54% of the total length of the hybrid assembly.

To identify the RAD markers, we sorted the trimmed reads to separate files according to barcode, removed the barcode sequence, and verified the restriction site sequence using an in-house Perl script named barcodesplitter5.3.pl (available in GitHub). The reads were then mapped to the hybrid assembly using the Bowtie2 tool (Langmead and Salzberg 2012). Polymorphic sites were filtered using the following criteria: (a) polymorphisms in parental fish were considered valid only if they were found present in fragments between 182 and 186 base pairs long (fragment extending to both sides from a restriction site) and had read coverage between 9 and 300 per base in both parents; (b) polymorphic sites were retained for linkage mapping when at least one of the parents was heterozygous, the polymorphism was biallelic in the offspring, and the offspring genotype distribution followed a Mendelian segregation pattern as tested by chi-square tests at 5% significance level following correction by false discovery rate according to the Benjamini and Hochberg approach (Benjamini and Hochberg 1995). Additionally, offspring were retained in the analysis if they had at least 1,000 markers genotyped with >8 read coverage, a criterion which resulted in removal of four offspring from the analysis. Polymorphic site filtering was completed using R (script “RADstats_final.R”; available in GitHub). The filtered markers were mapped to linkage groups and ordered using the Lep-MAP2 software (Rastas *et al.* 2016). Linkage between markers was accepted at LOD ≥ 9, upon which additional individual markers were added at LOD ≥ 7.

The linkage groups were initially constructed based on recombination frequencies and thereafter improved by testing the alternative ordering of markers using comparative mapping information from Atlantic salmon, a procedure hereafter referred to as salmonization. The latter was performed by first mapping the scaffolds from the European grayling hybrid assembly to the Atlantic salmon genome assembly (Lien *et al.* 2016), downloaded from the NCBI Genome database (RefSeq assembly GCF_000233375.1), using the nucmer tool in MUMmer 3.0 aligner (Kurtz *et al.* 2004). Prior to the alignment, Atlantic salmon chromosome sequences were repeat-masked using a salmon repeat database (ssal_repeats_v2.0) and RepeatMasker v4.0.3 (Smit *et al.* 2013–2015). The best matching position for each European grayling scaffold in the Atlantic salmon genome was determined by adding up the number of base pairs in each hit and the number of hits within a scaffold. Second, for each linkage group, markers mapping to the most frequently associated Atlantic salmon chromosome were included in the further salmonization procedure. At each step, the correct map was assumed to be the one with the shortest female map length calculated using Lep-MAP2 (Rastas *et al.* 2016). During the first step, the markers in each linkage group were initially reordered according to their locations in the Atlantic salmon assembly, and the resulting map lengths were calculated. In the second step, we investigated the salmonized European grayling linkage maps where breaks in the progression of map length increase indicated possible genomic rearrangements between the European grayling and Atlantic salmon genomes (Fig. S1). We then considered a portion of the largest breaks by applying either of the two following criteria: (a) the absolute map length difference of the break is > 10 map units for any of the markers or (b) the break length is at least eight times the standard deviation of that of all pairwise differences in adjacent markers in that linkage group. This step led to the identification of one to eight blocks of orderly progressed markers per linkage group. In the third step, we investigated the possibility of translocations from these blocks explaining our observations. To do this, we reconstructed each linkage group by permuting the order of the corresponding blocks and selecting the solution with the minimal length as the most parsimonious block order. To test the possibility of inversions in the most parsimonious block order, we then inverted each block and tested whether it further reduced the map length. At the end of this step we validated the final combination of rearrangements by testing if implementing all accepted changes indeed resulted in the minimal map length. The salmonization script “salmonize_final.R” is available in GitHub. As the final step, all maps were further manually curated with special attention given to regions that are known to have >90% sequence similarity in Atlantic salmon, namely, the pairs of Atlantic salmon chromosome arms including 2p-5q, 2q-12qa, 3q-6p, 4p- 8q, 7q-17qb, 11qa-26 and 16qb-17qa (Lien *et al.* 2016). European grayling linkage groups corresponding to salmon chromosome arm pairs 3q-6p, 7q-17qb and 11qa-26 mapped equally well to both of their Atlantic salmon homeolog counterparts. To identify the true homologs in these linkage groups, markers were aligned separately and ordered based on each of the Atlantic salmon homeologs using nucmer and LepMap2, and the best ordering homeolog was chosen as the linkage group identity. Linkage groups corresponding to Atlantic salmon chromosome arms 2q-12qa and 4p-8q had fused linkage maps that could not be separated.

#### Synteny-assisted genome scaffolding:

The European grayling scaffolds that contained markers in the final linkage groups and the scaffolds that had a MUMmer-alignment-based position in the Atlantic salmon genome were arranged into the final European grayling chromosomal order based on synteny with Atlantic salmon chromosomes unless there was strong evidence of a rearrangement based on the European grayling linkage map position. The alignment with Atlantic salmon was also used to orientate the scaffolds. Scaffolds were then concatenated into chromosome-level sequence assemblies by adding 100 base pair gaps between each adjacent scaffold.

### Repeat library construction and genome annotation

A comprehensive repeat library was built by combining *de novo* identified European grayling-specific repeats as well as repeat elements identified in the Atlantic salmon genome (available at: http://web.uvic.ca/grasp/salmon_v1.6). We initially ran the RepeatModeler software v. 1.0.11 (available at: http://www.repeatmasker.org/RepeatModeler; last accessed June 8, 2018) with default parameters. To compile a set of LTRs, we used the LTRharvest (Ellinghaus *et al.* 2008) and LTRdigest (Steinbiss *et al.* 2009) software as described in (http://weatherby.genetics.utah.edu/MAKER/wiki/index.php/Repeat_Library_Construction-Advanced; last accessed: June 8, 2018) and combined the results with the sequences identified by MGEscan-LTR (Lee *et al.* 2016). All the identified sequences were combined and filtered to remove redundancy. The resulting *de novo* set of sequences was combine-queried against the Universal Protein Resource database (UniProt proteins release 2017_08, Consortium 2017) to filter out any known proteins sequences. The remaining unclassified set of sequences was then annotated using RepeatClassifier, the Dfam database and TEclass (Abrusán *et al.* 2009).

An updated reference-based set of transcripts was constructed by first aligning the RNAseq reads to the improved assembly using STAR v. 2.6 (Dobin *et al.* 2013) followed by Cufflinks (Trapnell *et al.* 2010) for the prediction of transcript sequences. This along with the *de novo* assembled transcriptome described in Varadharajan *et al.* (2018) was used as an input to the PASA pipeline (Haas *et al.* 2003) to build a comprehensive transcript database.

Further, predictions from *ab initio* gene finders like SNAP (Korf 2004) and GeneMark-ES (Lomsadze *et al.* 2005) were also used as input into MAKER v. 2.31.9 (Cantarel *et al.* 2008). MAKER pipeline was run for two iterations with transcript evidence from PASA transcriptome assembly and protein coding sequences from the Atlantic salmon, GTF outputs from AUGUSTUS and GeneMark-ET resulting from BRAKER (Hoff *et al.* 2016), the UniProt database (UniProt proteins release 2017_08, Consortium 2017) as the protein evidence and the above described repeat library. MAKER was run with default options.

Functional annotation was added to the MAKER-predicted gene models using BLAST against UniProt database and domain information was added using InterProScan (Zdobnov and Apweiler 2001). MAKER-predicted gene models were then filtered based on Annotation Edit Distance (AED) and the presence of known PFAM domains to retain high confidence set of genes.

### Genome repetitiveness and repeat element assessment

Kmer repetitiveness of the previously published and current genome assemblies of European grayling, Atlantic salmon and rainbow trout (*Oncorhynchus mykiss*) were calculated using Jellyfish software v. 1.1.11 (Marçais and Kingsford 2011) using kmer size of 31. Repetitiveness was calculated by dividing counts of non-unique kmers by total kmers in the assembly. To investigate the European grayling genome in terms of repeat elements, the European grayling repeat library, containing 1,743 *de novo* repeats, was employed along with the repeats from RepBase v. 20.05 (Jurka *et al.* 2005). Transposable element sequences were curated by first detecting the host genes that were potentially of non-transposable element origin and then classifying the remaining transposable element sequences according to the classification system of Wicker *et al.* (2007). Transposable element abundances were estimated for both European grayling and Atlantic salmon. To remove from the final repeat analysis any repeats that potentially originated from host genes instead of transposable elements, the transposable element sequences were compared to two different repeat databases. These databases were the REPET-formatted RepBase v. 20.05 (Jurka *et al.* 2005) and the Swiss-Prot database available in UniProt (as of June 1, 2018). Comparisons were conducted by using the blastn (Altschul *et al.* 1990) and blastx (Gish and States 1993) algorithms with parameters set to *word_size = 7*, and to *e-value > 1×10^−10^*. A custom script named best_multi_blast_score_parser.pl (available in GitHub) was used to select the highest scoring hits for each potential transposable element sequence. Based on the best-scoring hits, each transposable element sequence was categorized as non-transposable element derived host gene and removed if it had a best-scoring hit to a Swiss-Prot sequence. The rest of the library hits were kept for further analysis.

To classify the transposable element sequences, they were compared to the RepBase repeats using both nucleotide sequence and protein similarity. To categorize transposable element sequences to class, order, and superfamily levels, the relevant information from RepBase was used in case a sequence had an acceptable alignment hit with this database. An alignment was accepted if it suggested high similarity between query and reference repeat, defined by Wicker *et al.* (2007). A high similarity alignment was at least 80 base pair long with at least 80% sequence similarity between query and reference repeat sequence, occupying at least 80% of the query repeat length (which we calculated after removing unknown nucleotides from the query sequence length). These thresholds concerned the blastn search. In case of a non-acceptable nucleotide alignment for a transposable element sequence, then this sequence was searched against the RepBase database using the blastx approach, with an alignment considered valid if the hit had *e-value < 1×10^−10^* (following Lien *et al.* 2016). The repeat element abundance in the European grayling and Atlantic salmon genomes was assessed for each chromosome separately using the RepeatMasker v. 4.0.7 tool (Chen 2004) by using the parameter -qq. The RepeatMasker-based locations of transposable element sequences in each of the two genomes were annotated with a script named “classifyGoodTEHits.R” (available in GitHub). The elements with marked difference in their abundance between the two genomes were sought out by using a linear model *log2(salmon abundancy+1) ∼ log2(grayling element abundancy+1)* using R (v. 3.4.0, R Core Team 2017) and elements that had residuals larger than 1.96 standard deviations from zero were considered outliers, that is, outside the 95% confidence interval limits.

#### Predicting centromere locations using the location of repeats:

Repetitive element content can reveal information about the chromosomal landscapes (Kaminker *et al.* 2002; Lien *et al.* 2016). To estimate the repeat content, copies of the generated repeat library were sought from the European grayling chromosomes using RepeatMasker. The abundances of different element classes were quantified using local regression for element abundancy over each chromosome with the R function lowess with parameter f = 0.2, and the maximum position for each element class in each of the European grayling chromosomes was extracted. These maximal density locations in each chromosome were analyzed using principal component analysis. Although centromeres are generally epigenetic structures that cannot be observed from the nucleotide order, some transposable elements have a tendency to accumulate in certain region of the genome (Daron *et al.* 2014). This has been previously observed in the Atlantic salmon genome (Lien *et al.* 2016) where Tc1-Mariner type elements were shown to accumulate in the centromeric regions. The chromosomal positions with the maximal abundancy of the two element classes, the centromere-related Tc1-mariner, and the most contrasting element class RTE-X were more closely inspected using the occurrences of each of the two element classes in 100 kilobase pair bins and local regression. Hypothetical centromere loci for each chromosome were predicted using the maximal estimates of the Tc1-Mariner-richness from the local regression curves. To predict the karyotype, the long:short arm ratio was estimated for each chromosome (following Levan, Fredga and Sandberg 1964). This was performed using the peak position of the Tc1-mariner element to calculate the length of the chromosomal fragments on both sides of the peak and dividing the longer length by the shorter one. Finally, the effect of chromosome size and karyotype on recombination frequencies was estimated using the linear model *map length ∼ chromosome size + long:short arm ratio*. To validate the effect of the long:short arm ratio in the full model, the chi-square test was performed to compare the full model to a reduced model with chromosome size as the only independent variable.

### Identification of the European grayling sex chromosome

The gene named *sdY* for sexually dimorphic on the Y-chromosome was searched using a tblastn (Altschul *et al.* 1990) homology search against the European grayling chromosome assembly. The rainbow trout *sdY* protein sequence (GenBank: BAM24747.1) was used as bait in this search. The RAD sequences were then remapped to the chromosome-level assembly and sex-biased loci were detected from the chromosomes.

### Comparison to the Northern pike genome

We compared the obtained European grayling chromosomes to the chromosomes of Northern pike (*Esox lucius*), a species that represents the closest sister group to Salmonids prior to the salmonid-specific whole genome duplication with an available genome assembly. The genome assembly was downloaded from the NCBI Genome database (RefSeq assembly GCA_000721915.1). Conserved synteny between European grayling and Northern pike was determined by aligning European grayling and Northern pike chromosome sequences using the nucmer tool in MUMmer 3.0 aligner (Kurtz *et al.* 2004) and keeping hits with identity ≥80.0 and matchcount ≥100. Homeologous European grayling chromosome pairs were named according to the Northern pike chromosome naming convention (Rondeau *et al.* 2014, Sutherland *et al.* 2016).

### Data availability

The PacBio reads, chromosome-level sequences and unmapped scaffolds over than 2000 base pairs have been deposited at NCBI SRA and Genomes under BioProject ID PRJNA464295. Scripts have been deposited to GitHub under the link https://github.com/tiinasa/graylinggenome. Supplemental material available at Figshare: https://doi.org/10.25387/g3.7728512.

## Results

### Chromosome-level European grayling genome assembly

#### Assembly of genomic scaffolds using long-read data:

By adding the PacBio long-read sequences to the published draft assembly of Varadharajan *et al.* (2018) and splitting 23 contigs that were determined as chimeric by initial comparison to the Atlantic salmon genome assembly, we obtained a 38% increase in N_50_ with a 24% decrease in L_50,_ a 62% increase in the maximum scaffold length, and a 16% increase in the total assembly length (TABLE 1). Altogether, the total number of scaffolds decreased by 25%, while the overall GC content remained almost identical at approximately 43% (TABLE 1).

**Table 1:** Assembly properties of different stages of the European grayling genome assembly process. Stage 1 represents the assembly built using only short-read DNA sequencing data (from Varadharajan *et al.* 2018). Stage 2 involves the outcome of the hybrid assembly process, which combined short- and long-read DNA sequencing data. Finally, stage 3 corresponds to the complete genome assembly that was produced using the linkage mapping data and synteny information with the Atlantic salmon genome. Numbers in brackets represent the percent increase/decrease over the previous stage for given statistics

Statistic	Stage 1	Stage 2		Stage 3	
N_50_	283,328	390,289	(38%)	33,018,251	(8,340%)
L_50_	1,359	1,030	(-24%)	20	(-98.5%)
N_90_	38,415	49,679	(29%)	23,618,429	(47,442%)
L_90_	6,620	5,397	(-18%)	40	(-99.4%)
Scaffolds	24,369	18,265	(-25%)	51	(-99.7%)
Length					
Total Average sd min max	1,468,519,221	1,575,987,192		1,485,210,005	
	60,261	86,285	(43%)	29,121,765	(33,651%)
	145,243	207,343		9,938,557	
	975	984	(1%)	6,483,087	(658,750%)
	2,502,076	4,048,953	(62%)	44,988,017	(1,011%)
Known bases	87%	95%		95%	
GC%	42.7%	42.8%		42.7%	

#### Linkage mapping:

RAD sequencing resulted in 7,684 informative SNP markers with a median female: male ratio of map distances at 1.75:1 (sd= 3.05). Postfiltering, 6,076 markers were assigned to the final linkage groups (TABLE S1). The final female-based total map length was 3,044 centi-Morgan (cM) (Fig. S2 and TABLE S2). The average female map length per million chromosomal base pairs was 2.0 map units (TABLE S1).

#### Synteny-assisted genome chromosome building:

**Figure 1:**
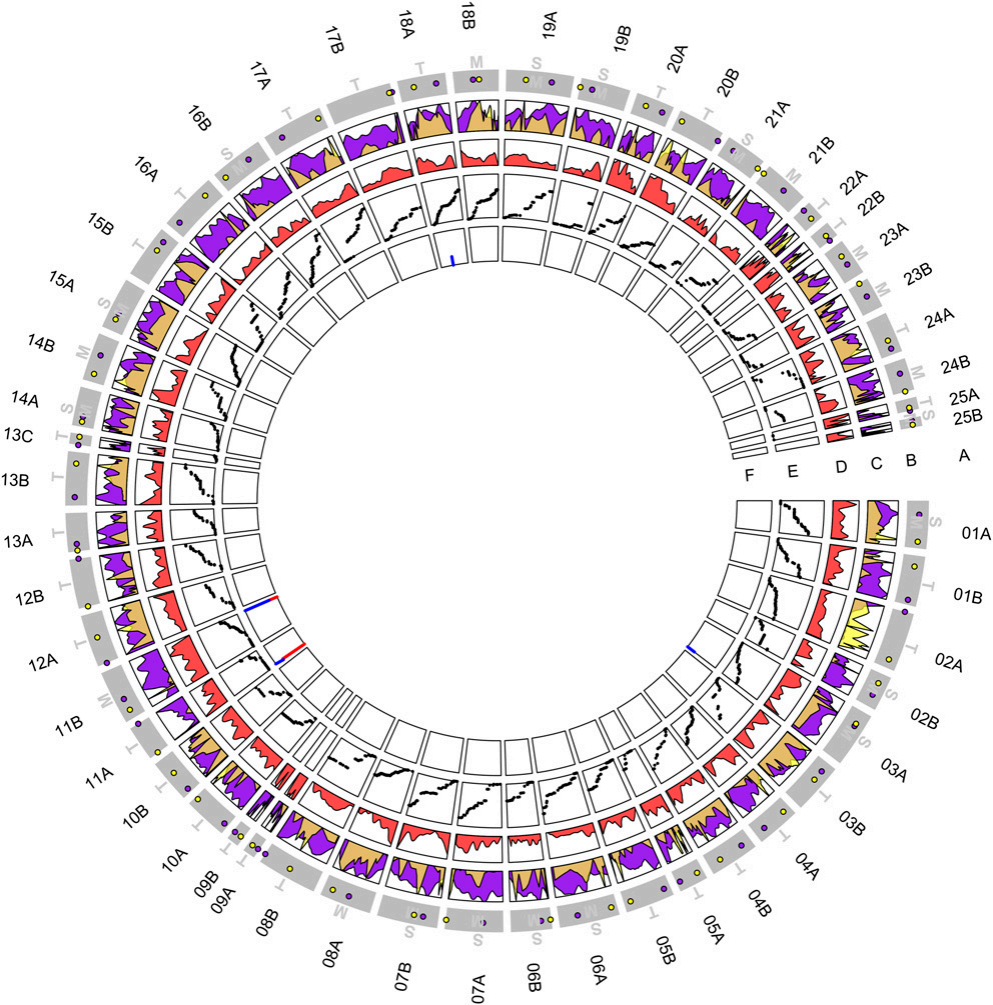
The European grayling genome. The tracks indicate the following information: (A) Chromosome number according to Northern pike, used as a non-WGD outgroup, and predicted chromosomal type (M = metacentric; SM = submetacentric; and T = acrocentric); (B) Predicted centromere (in purple) and telomere (in yellow) locations; (C) Proportions of the Tc1-mariner (in purple) and RTE-X elements (in yellow), which are used to predict centromere and telomere positions, respectively; (D) Gene density; (E) Female linkage map units; and (F) Female- (in red) and male-biased (in blue) sex-biased loci with the *sdY* gene region indicated.

The synteny-assisted chromosome building step represented the biggest improvement in the genome assembly process (TABLE 1). Together with the linkage mapping information, we managed to reconstruct all 51 European grayling chromosomes (Figure 1). Nevertheless, of the total of 18,265 scaffolds from the hybrid stage of the assembly, a large number of relatively small-sized scaffolds were left unassigned (N = 8,938 scaffolds ranging in length from 984 to 1,162,211 base pairs of which N = 3,780 scaffolds with > 2,000 base pairs length are available at NCBI), which corresponded to a total length of 91,704,787 unassigned base pairs (or 5.8% of the total genome assembly length).

### Genome repetitiveness and repeat element assessment

The repetitiveness of identical sequences in the chromosome-level European grayling assembly was estimated at 11.9%. By comparison, we estimated repetitiveness at 29.3% in the Atlantic salmon genome and 9.8% in the reported genome assembly of the rainbow trout using the same software. The RepBase and Swiss-Prot searches resulted in a best-scoring hits list including 1,090 transposable element sequences, of which 32 potential host genes were removed from further transposable element analysis. Of the remaining transposable element sequences, 287 had nucleotide-based matches and 434 had protein-based matches to RepBase after filtering. After the matches were combined, they contained 586 unique transposable element sequences. These classified transposable element sequences covered 83% of the European grayling repeat sequences. Altogether, 47.4% of the European grayling genome assembly and 52.3% of the Atlantic salmon genome assembly were covered by these repeats, which could be distinguished into 24 superfamilies of transposable elements (TABLE 2). Of the repeat elements identified, 14 were only found in the Atlantic salmon genome assembly, while only three were more abundant in the European grayling genome assembly (TABLE S3 and Figure 3).

**Table 2:** Transposable element classification and abundances in the European grayling and Atlantic salmon genomes

Transposable element			European grayling		Atlantic salmon	
Class	Order	Superfamily	base pairs	% coverage	base pairs	% coverage
RNA-transposons (class I)	LINE	Jockey	133311368	9.0	228445871	10.2
		RTE	10629973	0.7	12021858	0.5
		L1	6987652	0.5	15019466	0.7
		I	1925092	0.1	2994056	0.1
	LTR	Gypsy	88958008	6.0	120880877	5.4
		ERV	23336303	1.6	29951691	1.3
		Bel-Pao	4559403	0.3	5228163	0.2
		Copia	1009467	0.1	3887925	0.2
	SINE	tRNA	6099534	0.4	12208774	0.5
		Unknown	439123	0.0	1325090	0.1
	DIRS	DIRS	6352447	0.4	13786775	0.6
	PLE	Penelope	1392065	0.1	1728304	0.1
	Unknown	Unknown	102556	0.0	157518	0.0
DNA-transposons (class II)	TIR	Tc1-Mariner	143125085	9.6	226246051	10.1
		hAT	18745228	1.3	26354108	1.2
		CACTA	1063048	0.1	1575430	0.1
		PIF-Harbinger	606932	0.0	788870	0.0
		PiggyBac	237022	0.0	555705	0.0
	Unknown	Unknown	5627491	0.4	90211317	4.0
		Sola	148327	0.0	373468	0.0
		Ginger1	59568	0.0	66177	0.0
		ISL2EU	37185	0.0	0	0.0
	Crypton	Crypton	1763736	0.1	2838181	0.1
	Maverick	Maverick	4732	0.0	147407	0.0
Unknown			247405186	16.7	374107217	16.7
**Total repeat coverage**			**703926531**	**47.4**	**1170900299**	**52.3**

#### Predicting centromere locations using the location of the repeats:

We considered the Tc1-Mariner abundance to peak around the centromeric regions (Figure 1). A LINE-class RTE-X retrotransposon was also found located the furthest from Tc1-Mariner excluding some unknown and simple repeat categories, suggesting a potential subtelomeric enrichment (Fig. S3). Using the Tc1-Mariner abundances we predicted 29 telocentric and 22 sub(metacentric) karyotypes (Figure 1). After correcting for the chromosome size in megabases, the long:short arm ratio had a negative correlation with the female map length (estimate -1.83, *P* = 0.0335, F(2,45)=26.94, adjusted R-squared = 0.5246 for the whole model). The chi-square test confirmed that the long:short arm ratio was indeed a significant variable (*P* = 0.0283) when predicting the map length of a chromosome. This result suggests that the metacentric chromosomes have a relatively higher recombination rate than comparably sized telocentric chromosomes.

#### Genome rearrangements between European grayling and Atlantic salmon:

**Figure 2:**
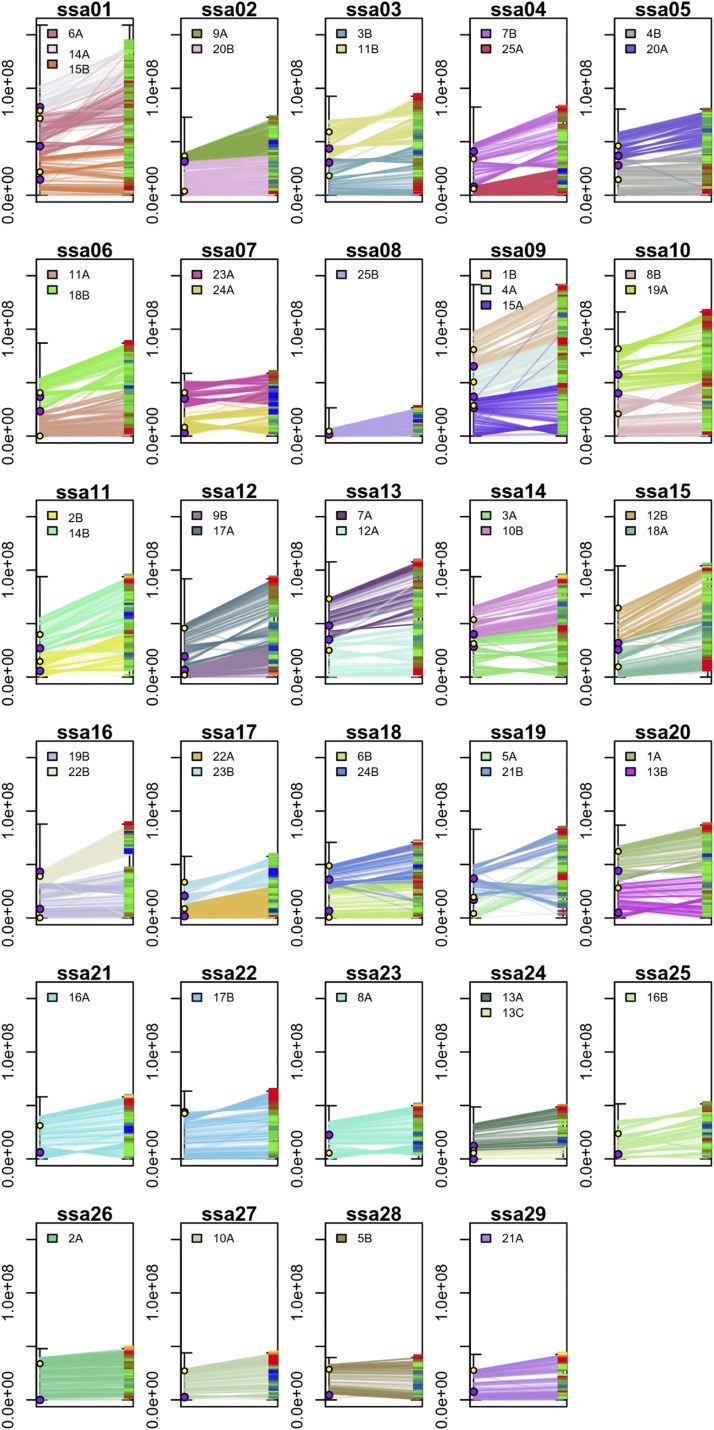
Chromosomal rearrangements between the European grayling and Atlantic salmon genomes. Each box represents an Atlantic salmon chromosome depicted on the right side of the box. The corresponding European grayling chromosomes are shown in different colors on the left side. Horizontal lines represent corresponding positions between the two genomes, with sequence identity depicted in a bluered scale on the right side of the box. Purple and yellow circles depict predicted centromere and telomere positions in the European grayling chromosomes, respectively. The axes scales represent the log_2_-transformed abundance in base pairs +1.

Rearrangements between European grayling and Atlantic salmon chromosomes suggested conservation of the synteny within blocks of chromosome arms in both of the species, but with frequent chromosomal inversions observed between the two genomes. In particular, we detected 119 blocks from which we could confidently interpret 18 as pericentric and 24 as paracentric inversions in European grayling (Figure 2, TABLE S4, and Fig. S4). Compared to Atlantic salmon, in which many chromosomal fusions have occurred after the tetraploid salmonid ancestor, the ancestral chromosome identities were conserved in European grayling with the exception of one chromosomal fission that was noticed (Figure 2).

### Identification of the European grayling sex chromosome

The *sdY* locus was mapped to 11A [2,137,039-2,136,679]; (*e-value = 1.74E-54*, score = 190). After remapping the RAD reads, we found the sex-biased loci to be clearly enriched at European grayling 11A close to the *sdY* locus and in the 11B homeolog, with four sex-biased loci found on 11A and four on 11B. Additionally, three sex-biased loci were more randomly distributed in the genome, more specifically on chromosomes 3A and 18A and in the scaffold Contig7739 (Figure 1).

### Comparisons with the genome of Northern pike

The European grayling chromosomes could be matched to the Northern pike counterparts in a 2:1 ratio except for one ancestral-duplicated chromosome that had been subsequently split in two in European grayling (13A and 13C) (TABLE S1). European grayling chromosomes were named according to the corresponding pike orthologs (TABLE S1).

## Discussion

By assembling the European grayling genome up to the level of chromosomes and comparing it with that of Atlantic salmon, we provided some novel insights about the very distinct genome evolution processes that have been ongoing in European grayling (Phillips and Ráb 2001) and, in particular, we reported on the complete absence of chromosomal fusions and the somewhat frequent occurrence of chromosomal inversions (Figure 2). The absence of a fusion event in European grayling could be parsimoniously hypothesized by observing that the European grayling homeologue chromosomes always mapped to single Northern pike chromosomes (TABLE S1). This finding suggests the absence of chromosomal fusions in either of these two genomes since the last whole genome duplication event. Notably, a single case of chromosomal fission was observed behind the generation of European grayling chromosomes 13A and 13C (TABLE S1). The absence of fusions agrees with previous hypotheses based on karyotype information (Phillips and Ráb 2001) and is confirmed for the first time at the sequence level. The relatively frequent occurrence of chromosomal inversions in the European grayling lineage was also confirmed by identifying at least 51 inversions between the European grayling and Atlantic salmon genomes, which covered as much as 45% of the total length of the European grayling genome assembly (TABLE S4). Detailing the origin of these inversions, whether specific to European grayling or to Atlantic salmon, proved to be a challenging task. Comparisons of the order of available markers between the genomes of these two species and that of Northern pike are often problematic due to the loss of synteny within chromosome arms and smaller number of European grayling scaffolds reliably positioned in the Northern pike genome (Fig. S4). Nevertheless, for nine of the inversions, it was clear that six were specific to the European grayling genome and three were specific to the Atlantic salmon genome (TABLE S4). Thus, chromosomal inversions appear to have played a role in the genome evolution process in both of these species, albeit more frequently identified in the European grayling genome. A relatively large number of the recognized inversions, 18 out of the 42 resolved cases, were also found to be pericentric, that is, inversions that included the centromere (TABLE S4). Taken together, these findings may also explain the relatively large number of chromosomal arms observed in European grayling, as a pericentric inversion of the assumedly acrocentric ancestral chromosome would double the number of chromosomal arms (Phillips and Ráb 2001).

Transposable elements may have played a key role in genome evolution processes (Auvinet *et al.* 2018). Additionally, these elements may be important in the rediploidization process by generating sequence divergence that would separate the homeologs. In particular, the comparison of transposable element classes between organisms with very different genomic rearrangements, such as between European grayling and Atlantic salmon, may be of interest. We found that retrotransposons (class I transposable elements) are more abundant compared to DNA transposons (class II transposable elements) with 1.7 times and 1.3 times higher abundance in the European grayling and Atlantic salmon genomes, respectively (TABLE 2). This is similar to what has been observed in the genomes of a wide variety of other eukaryotes, such as many plants, insects, amphibians, reptiles, and mammals, which were found to have a relatively higher proportion of retrotransposons than DNA transposons (reviewed in Canapa *et al.* 2015). Nevertheless, this is different from what has been found in many non-salmonid fish, which were found to have DNA transposons as the most abundant class (Canapa *et al.* 2015). The differential accumulation of transposable elements between lineages may be playing a significant role in genome evolution processes, but unraveling the complexity of underlying reasons behind such differences could not be investigated in this study.

**Figure 3:**
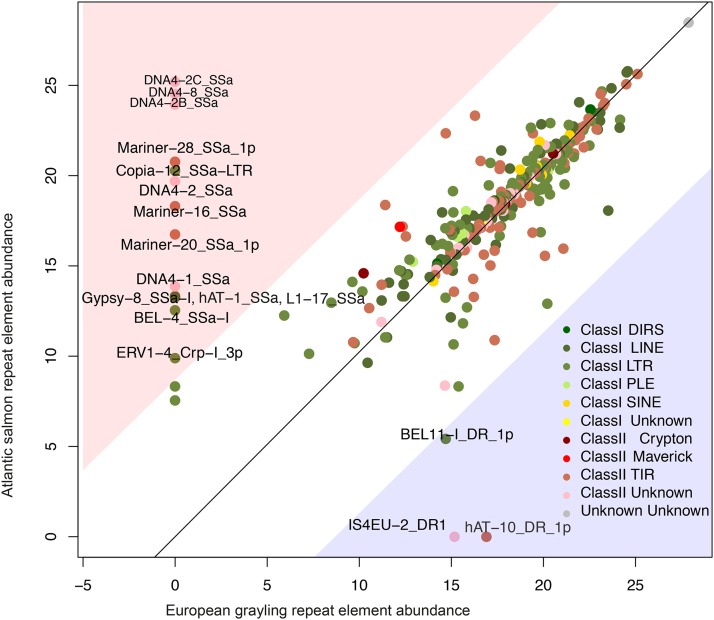
Comparison of repeat element abundances in the European grayling and Atlantic salmon genomes. The RepBase names of the elements that were more abundant in either of the two species and outside the 95% confidence limit are also given. Residuals are colored to indicate element class and order categories.

Additionally, the comparison of abundances of the transposable elements between European grayling and Atlantic salmon resulted in the recognition of 14 Atlantic salmon-specific and three European grayling-specific transposable elements (Figure 3 and TABLE S3). The Atlantic salmon-specific elements include the DNA transposons DNA4-1, DNA4-2, DNA4-2B, DNA4-2C and DNA4-8, which altogether covered 80 megabase pairs (3.57%) in the Atlantic salmon genome, but were found to be completely absent in European grayling (TABLE S3). The DNA transposons Mariner-16, Mariner-20, and Mariner-28 were also found to be Atlantic salmon-specific (Figure 3 and TABLE S3). These elements belong to the same Tc1-Mariner superfamily, which represents one of the most abundant categories of transposable elements in salmonids, with a major suspected role in the Atlantic salmon rediploidization process (Lien *et al.* 2016). Another case of an Atlantic salmon-specific element is the Copia-12 retrotransposon (Figure 3 and TABLE S3), which belongs to the Copia superfamily of retrotransposons that was recently suggested to have a role in chromosomal diversification and speciation in other teleost fishes (Auvinet *et al.* 2018). Among the European grayling-specific elements, hAT-10 from the hAT DNA transposon superfamily covered 123,702 base pairs (0.01%) of the assembly and was completely absent in the Atlantic salmon genome assembly (Figure 3 and TABLE S3). The hAT DNA transposons, such as the Tc1-Mariner ones, are so-called cut-and-paste elements that have transposition mechanisms with the potential to actively induce genomic rearrangements in addition to indirect ways to generate homologous recombination of element copies. The accumulation of a particular transposable element in one of the two species may be considered as an indication of lineage-specific transposable element activity. These findings may provide unique insights to stimulate further research aimed at better understanding the molecular drivers of these distinct genome evolution processes. While highly accurate in repeat identification and suitable for our purpose to simply compare the element abundances between European grayling and Atlantic salmon, conventional software such as RepeatMasker that we applied has been reported to under-estimate the abundances of transposable elements (de Koning *et al.* 2011). Future studies could benefit from using more sensitive approaches such as repetitive sequence clustering (de Koning, *et al.* 2011) as they may enable improved estimation of repeat element abundances. Moreover, they may allow further insight into repeat community structure and key element identification using network approaches (Wacholder *et al.*, 2014; Levy *et al.* 2017) thus enabling more detailed investigations of the repeat element proliferation dynamics among salmonids.

**Figure 4:**
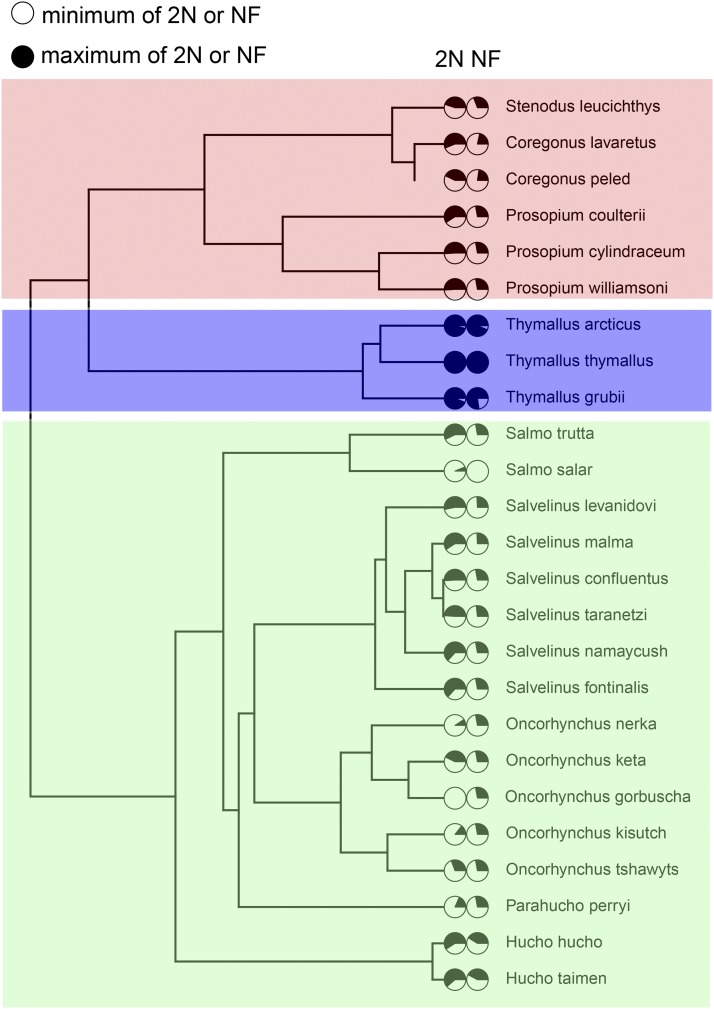
Karyotypic changes among salmonid taxa. A Bayesian chronogram tree based on mitochondrial sequence (tree obtained and edited from Shedko *et al.* (2013), doi: 10.5061/dryad.r42qf) of those salmonids that have diploid chromosome number (2n) and the number of chromosome arms (NF) available in Phillips and Ráb (2001).

Based on current knowledge we can only speculate what may be the role of the distinct European grayling chromosome architecture in the evolution of the species. Qumsiyeh (1994) hypothesizes that high diploid chromosome number leads to increased recombination rates, which in the case of the freshwater European graylings, may be associated with low differentiation, an advantageous trait in variable freshwater environments (Phillips and Ráb 2001) (Figure 4). In contrast, reduction of chromosome numbers in the other salmonid lineages may be associated with anadromous life history strategy (Phillips and Ráb 2001). It has been suggested that periods of relaxed purifying selection, as in bottlenecked populations, may be necessary for the deleterious effects of chromosomal rearrangements to become fixed (Lynch and Conery 2003; Lynch 2007). While possibly initially stochastic in nature, the resulting effects of chromosome evolution on mutation and recombination rates can result in directed evolution (Lynch 2007) and phenotypic change. Also, following gene duplication, lineage-specific loss of duplicated gene copies (Lynch and Conery 2000) or possibly divergent expression evolution such as observed between European grayling and Atlantic salmon (Varadharajan *et al.* 2018), may contribute to speciation. Evidence of distinct genome evolution processes may provide avenues for further research aimed at exploring links between life history differences in salmonids and the evolution of distinct genome architectures. Transposable element activity, with lineage-specific differences such as those observed between European grayling and Atlantic salmon, is a major driver of genome evolution (Kazazian 2004) and may have been also involved in the distinct genome evolution processes observed here. Furthermore, chromosomal inversions, such as those found frequently in the European grayling genome, have been suggested to have profound effects in the adaptation and speciation processes (Wellenreuther and Bernatchez 2018). For instance, they have been reported to increase genome sequence divergence between marine and freshwater ecotypes of a stickleback species *Pungitius pungitius* (Nelson and Cresko 2018) as well as between nonmigratory and migratory ecotypes of Atlantic cod (*Gadus morhua*) (Berg *et al.* 2016; Kirubakaran *et al.* 2016). Computer simulations supported these observations and showed that chromosomal inversions may accelerate speciation particularly in certain conditions, such as when adaptation involves multiple genes with small individual fitness effects (Feder *et al.* 2014). Experimentation in house mouse (*Mus musculus domesticus*) has additionally demonstrated the possibility of rapid divergence mediated by Robertsonian fusions (Garagna *et al.* 2014). We anticipate that further salmonid-centric research in this direction aided by help from the chromosomal-level European grayling assembly that we provide will illuminate several open questions that stem from these observations.

Cases of residual tetrasomy and elevated sequence similarity between homeologous chromosomes have been reported in many salmonids, suggesting that some rediploidization in these salmonid species may be ongoing (Lien *et al.* 2016). Although some species-specific differences in the residually tetrasomic regions have been reported, the tetraploid state appears to be conserved among salmonid species in seven to eight homeologous chromosome pairs (as summarized in Sutherland *et al.* 2016). Although otherwise distinctive, the karyotype evolution of European grayling was comparable to that of most salmonids in the case of residually tetrasomic regions (in chromosomes 9A & 9B homologous to ssa02q & ssa12qa, respectively and 25A & 25B homologous to ssa04p & ssa08q, respectively) also being observed in the European grayling genome assembly based on shared linkage maps. Similarly, other regions (in chromosomes 2A & 2B homologous to ssa26 & ssa11a, respectively; 11A & 11B homologous to ssa6a & ssa3b, respectively; 20A homologous to ssa5b; and 23A & 23 B homologous to ssa7b and 17b, respectively) with reoccurring residual tetrasomy reported among salmonids (Sutherland *et al.* 2016) had elevated sequence similarity, which has also be used as a predictor for recent or ongoing tetrasomy (Lien *et al.* 2016) (TABLE S1). Residual tetrasomy appears to have persisted in both Salmoninae and Thymallinae since the two lineages split, though the pace of the remaining rediploidization has been very slow since the lineage diversification (Lien *et al.* 2016). The evolutionary significance of persistent residual tetrasomy remains unknown, but the existence of residual tetrasomy in the ancestral-like European grayling genome suggests that tetrasomy would be independent of chromosomal fusions typical of other salmonids (Phillips and Ráb 2001; Lien *et al.* 2016) and instead be favored by some other factor. The majority of the sex-linked loci detected were found in the homeologous European grayling chromosome pair 11. Additionally, we located the *sdY* gene, reported as male-specific among many other salmonids (Yano *et al.* 2013), in chromosome 11A; thus, we concluded that chromosome 11A is the European grayling sex chromosome.

In conclusion, by utilizing the novel resource of a chromosome-level genome assembly for European grayling, we were able to make some intriguing observations about the genome evolution processes in salmonids that confirmed previous hypotheses and generated new questions. We confirmed the absence of chromosomal fusions and the somewhat high abundance of pericentric inversions in European grayling and highlighted the differences compared to the large number of chromosomal fusions that have taken place in the Atlantic salmon. We also highlighted novel and already described instances of transposable elements with a role in driving these different genome evolution processes. We further identified similar homeologous regions under residual tetrasomy in European grayling as in the genomes of many other salmonid species and discussed the potential underlying evolutionary causes behind the distinctive karyotype evolution of Thymallinae among salmonids as well as the role of genomic rearrangements in speciation. We anticipate that as more salmonid genomes are sequenced, many of these questions will be further investigated and advance our understanding of the major molecular mechanisms that have shaped the salmonid genomes since their last common whole genome duplication event.
